# Concomitant medication of cetirizine in advanced melanoma could enhance anti-PD-1 efficacy by promoting M1 macrophages polarization

**DOI:** 10.1186/s12967-022-03643-w

**Published:** 2022-09-30

**Authors:** Domenico Mallardo, Ester Simeone, Vito Vanella, Maria Grazia Vitale, Marco Palla, Luigi Scarpato, Miriam Paone, Teresa De Cristofaro, Valentina Borzillo, Alessio Cortellini, Francesca Sparano, Sandro Pignata, Francesco Fiore, Corrado Caracò, Piera Maiolino, Antonella Petrillo, Ernesta Cavalcanti, Secondo Lastoria, Paolo Muto, Alfredo Budillon, Sarah Warren, Paolo Antonio Ascierto

**Affiliations:** 1 Melanoma, Cancer Immunotherapy and Development Therapeutics Unit, Istituto Nazionale Tumori – IRCCS – Fondazione ”G. Pascale”, Naples, Italy; 2 Radiation Oncology Unit, Istituto Nazionale Tumori – IRCCS –Fondazione ”G. Pascale”, Naples, Italy; 3 Department of Surgery & Cancer, Imperial College London, Hammersmith Hospital, Du Cane Road, London, W120HS UK; 4 Department of Urology and Gynecology, Istituto Nazionale Tumori – IRCCS –Fondazione ”G. Pascale”, Naples, Italy; 5 Interventional Radiology Unit, Istituto Nazionale Tumori – IRCCS –Fondazione ”G. Pascale”, Naples, Italy; 6 Division of Surgery of Melanoma and Skin Cancer, Istituto Nazionale Tumori – IRCCS – Fondazione ”G. Pascale”, Naples, Italy; 7 Hospital Pharmacy, Istituto Nazionale Tumori – IRCCS – Fondazione ”G. Pascale”, Naples, Italy; 8 Radiology Division, Istituto Nazionale Tumori – IRCCS – Fondazione ”G. Pascale”, Naples, Italy; 9 Division of Laboratory Medicine, Istituto Nazionale Tumori – IRCCS –Fondazione ”G. Pascale”, Naples, Italy; 10 Nuclear Medicine Unit, Istituto Nazionale Tumori – IRCCS – Fondazione ”G. Pascale”, Naples, Italy; 11 Experimental Pharmacology Unit, Istituto Nazionale Tumori – IRCCS – Fondazione ”G. Pascale”, Naples, Italy; 12 NanoString Technologies, Seattle, WA USA

**Keywords:** Malignant melanoma, Anti-PD-1, Cetirizine, Tumor-associated macrophage, Drug repurposing

## Abstract

**Background:**

The clinical observation showed a potential additive effect of anti-PD-1 agents and cetirizine in patients with advanced melanoma.

**Methods:**

Clinical outcomes of concomitant cetirizine/anti-PD-1 treatment of patients with stage IIIb–IV melanoma were retrospectively collected, and a transcriptomic analysis was performed on blood samples obtained at baseline and after 3 months of treatment.

**Results:**

Patients treated with cetirizine concomitantly with an anti-PD-1 agent had significantly longer progression-free survival (PFS; mean PFS: 28 vs 15 months, HR 0.46, 95% CI: 0.28–0.76; p = 0.0023) and OS (mean OS was 36 vs 23 months, HR 0.48, 95% CI: 0.29–0.78; p = 0.0032) in comparison with those not receiving cetirizine. The concomitant treatment was significantly associated with ORR and DCR (p < 0.05). The expression of *FCGR1A/CD64*, a specific marker of macrophages, was increased after the treatment in comparison with baseline in blood samples from patients receiving cetirizine, but not in those receiving only the anti-PD1, and positively correlated with the expression of genes linked to the interferon pathway such as *CCL8* (rho = 0.32; p = 0.0111)*, IFIT1* (rho = 0.29; p = 0.0229)*, IFIT3* (rho = 0.57; p < 0.0001)*, IFI27 (*rho = 0.42; p = 0.008), MX1 (rho = 0.26; p = 0.0383) *and RSAD2* (rho = 0.43; p = 0.0005).

**Conclusions:**

This retrospective study suggests that M1 macrophage polarization may be induced by cetirizine through the interferon-gamma pathway. This effect may synergize with the immunotherapy of advanced melanoma with anti-PD-1 agents.

**Supplementary Information:**

The online version contains supplementary material available at 10.1186/s12967-022-03643-w.

## Key messages

What is already known on this topic—Macrophage-based immunotherapy has been developed in the last decade as a novel strategy based on the modulation of this population in the tumor microenvironment, and molecular mechanisms required to induce the tumoricidal polarization of TAMs could be of therapeutic relevance.

What this study adds—M1 macrophage polarization may be induced by cetirizine and that this effect may synergize with. The immunotherapy of advanced melanoma with anti-PD-1 agents.

How this study might affect research, practice or policy—patients with advanced melanoma may have better outcomes if immunotherapy with anti PD-1 was associated with cetirizine.

**Supplementary Information:**

The online version contains supplementary material available at 10.1186/s12967-022-03643-w.

## Background

The availability of immune checkpoint inhibitors, specifically anti-CTLA-4 and anti-PD-1 agents, has radically changed the prognosis of patients with advanced melanoma. Indeed, PD-1 inhibitors are currently considered the standard of care as adjuvant therapy in high-risk resected stage III or IV melanoma, and tumor regression and long-term durable cancer control are possible in a high proportion of patients, compared with < 10% before the introduction of immunotherapy [3, 14]. Compared with monotherapies, even higher response rates and longer survival were obtained with the combination of anti-CTLA-4 and anti-PD-1. The median OS was 72.1 months with the combination of nivolumab and ipilimumab, with a 57% OS rate at 6.5 years in patients with *BRAF*-mutant tumors and 46% in those with *BRAF* wild-type tumors [19]. Nevertheless, unmet needs still exist with many patients who do not profit from the current standard of care. Therefore, further treatment strategies are necessary to address primary or acquired resistance to treatments. In recent years, non-oncology drugs are gaining mounting attention for their potential anti-cancer activities, pending a mechanistic rationale for their use exists [15, 17, 21]. Beta adrenergic blocking drugs were found to have potential synergy with anti-PD1 agents [5].

Macrophage-based immunotherapy has been developed in the last decade as a novel strategy based on the modulation of this population in the tumor microenvironment [2, 13, 18]. Tumor-associated macrophages (TAM) have a role in tumor development, chemoresistance, immune evasion and metastasis [2]. TAMs are known to either promote or suppress tumor growth depending on their activation status, and macrophages with killing or antitumor activity are indicated as M1 (or classically activated). In contrast, tumor-promoting or -healing macrophages are named M2 (or alternatively activated) (Mosser 2008) [11]. The depletion of TAMs has been mainly investigated as a therapeutic strategy, aiming at counteracting the tumor growth promotion of M2 TAMs [13], but some studies aimed at reprogramming TAMs toward a tumoricidal M1 phenotype (Beatty 2011; Kaneda 2016; Guerriero 2017) [11]. Indeed, molecular mechanisms required to induce the tumoricidal polarization of TAMs could be of therapeutic relevance. IFN-γ, previously identified as macrophage-activating factor (MAF), has a major role in regulating macrophage activity toward a tumoricidal activity. However, its interactions with second signals in the tumor microenvironment, such as cytokines and killed bacteria, are not completely clarified [1, 10, 11]. IFN-γ production was found to be significantly increased following 4 weeks of therapy with cetirizine, one of the most commonly used antihistamines for treating allergic diseases. In addition, cetirizine induced a shift in the Th1/Th2 cytokine balance toward a Th1 type response by increasing IFN-γ production [16]. In mice, antihistamine treatment delayed colorectal cancer development and enhanced immune response induced by cytokines [9]. Based on this evidence, it is possible to speculate that concomitant use of cetirizine with anti-PD-1 agents could promote tumoricidal polarization of TAMs and thus enhance immunotherapy efficacy.

This study aims to better understand the mechanisms involved in the activation of macrophages towards an antitumor M1 phenotype in patients with advanced melanoma, based on the clinical observation of a potential additive effect of anti-PD-1 agents and cetirizine.

## Patients and methods

### Study design

A retrospective study was carried out in the Istituto Nazionale Tumori – IRCCS – Fondazione "G. Pascale," Naples, Italy, upon communication to the local Ethical Committee [protocol n.17/17 oss]. The study was performed in accordance with the revised version of the declaration of Helsinki (52^nd^ WMA General Assembly, Edinburgh, Scotland, October 2000).

Consecutive adult patients with metastatic melanoma at unresectable stage IIIb–IV and histologically confirmed, treated with an anti-PD-1 agent, either in the first line or pretreated with ipilimumab,, aged over 18 years were enrolled between July 2014 and July 2018. Concurrent use of cetirizine was retrospectively ascertained. All patients provided their written informed consent.

### Evaluation of outcomes

RECIST 1.1 criteria were used to evaluate the tumor response as complete response (CR), partial response (PR), stable disease (SD), or progressive disease (PD). The following parameters were recorded: concurrent use of cetirizine, response rate at first assessment, progression-free survival (PFS; defined as the time from the administration of the first dose of anti-PD-1 agent to documented radiological progression, death or lost to follow-up, whichever occurred first), overall survival (OS; defined as the time from the administration of the first dose of anti-PD-1 agent to death or lost-to-follow-up, whichever occurred first), disease control rate (DCR; defined as the sum of CR, PR, and SD > 1 year), objective response rate (ORR; defined as the sum of CR and PR), Eastern Cooperative Oncology Group Performance Status (ECOG PS), history of allergy, American Joint Committee on Cancer (AJCC) distant metastases category (M), lactate dehydrogenase (LDH) level.

### Transcriptomic analysis

To conduct a gene profile analysis, blood samples from enrolled patients were collected at baseline and after 3 months of treatment (concurrently with the first assessment) with an anti-PD-1 agent or at discontinuation if occurring before 3 months. RNA from whole blood was extracted using RNA blood mini-Kit (Qiagen). Purified RNA was used for hybridization and subjected to gene profiling analysis on NanoString nCounter through PanCancer IO 360 panel, characterized by 770 human genes involved in the interplay between tumor microenvironment and immune response. Gene data were normalized using nSolver Version 4.0 Software; NanoString. Counts were normalized to External RNA Controls Consortium (ERCC) technical controls and 30 housekeeping genes.

### Statistical analysis

Demographic and clinical data were tabulated using descriptive statistics. PFS was calculated from the start of treatment with anti-PD-1 to the evidence of progressive disease or death, whichever occurs first; OS was calculated from the start of treatment with anti-PD-1 to death or censored at the last follow-up. Survival times were analyzed using the Kaplan–Meier method, and differences among curves were assessed by the log-rank test. Using a Cox regression model, hazard ratios (HRs) and their 95% CIs were estimated. Spearman's rho analysis and χ^2^ log test were used to evaluate the association of variables.

## Results

### Patients' characteristics and outcomes

Overall, 121 patients were enrolled, of whom 53 (43.8%) were females, with a median age of 63 years (range: 27–91 years). The stage according to the AJCC VII classification was IV in 115 (95%) patients, IIIC in 5 (4.2%), and IIIB in 1 (0.8%). Baseline characteristics are reported in Table 1. Radiotherapy had not been used in any patient.

**Table 1:** Patients' characteristics at baseline

Patient characteristics	Anti-PD1 + Cetirizine (n = 71) n (%)	Anti-PD1 alone n = 50, n (%)
Age (years), mean (range)	61 (27–90)	64 (38–91)
Gender (female/male)	30 (42.3)/41 (57.7)	23 (43.8)/27 (56.2)
*Melanoma AJCC VII stage*		
• Stage IV	67 (94.4)	47 (94.0)
• Stage IIIC	3 (4.2)	2 (4.0)
• Stage IIIB	1 (1.4)	1 (2.0)
CNS metastases at baseline	14 (19.7)	14 (28.0)
*BRAF status*		
• Wild-type	56 (78.9)	35 (70.0)
• Mutation	10 (14.1)	12 (24.0)
• NA	5 (7.0)	3 (6.0)
*Type of anti-PD-1 agent*		
• Pembrolizumab	25 (35.2)	18 (36.0)
• Nivolumab	46 (64.8)	32 (64.0)
*Line of treatment (anti-PD-1)*		
• First-line treatment	49 (69.0)	39 (78.0)
• Second-line treatment	22 (31.0)	11 (22.0)
*Type of previous therapy*		
• Ipilimumab	22 (100)	11 (100)
*Response rate at first assessment*		
• Complete response	8 (11.3)	3 (6.0)
• Partial response	16 (22.5)	8 (16.0)
• Stable disease	24 (33.8)	9 (18.0)
• Progression disease	23 (32.4)	30 (60.0)
*ECOG PS*		
• 0/1	63 (88.7)	40 (80.0)
• > 2	8 (11.3)	10 (20.0)
Allergic patients	4 (5.7)	5 (10.0)
Drugs related allergies	3 (4.2)	4 (8.0)
Sseasonal allergies	1 (1.4)	1 (2.0)
Median OS (months)	27.59	19.30
Median PFS (months)	18.05	10.02
*M category*		
• M0	4 (5.6)	2 (4.0)
• M1a	9 (12.7)	7 (14)
• M1b	9 (12.7)	7 (14.0)
• M1c	49 (69.0)	34 (68.0)
*LDH*		
• High	22 (31.0)	17 (34.0)
• Normal	25 (35.2)	23 (46)
• NA	24 (33.8)	10 (20)

In all patients who had received it, cetirizine had been used as a premedication on the day of immunotherapy (10 mg once); only 9 patients were allergic (Table 1). The patients who had not received cetirizine had not been administered any other antihistamine in the previous month. Cetirizine treatment was used concomitantly with anti-PD-1 in 71/121 patients, in 49/88 patients naïve to checkpoint inhibitors (named naïve thereafter), and in 22/33 patients pretreated with ipilimumab (named pretreated thereafter). The two groups, with and without cetirizine, were balanced for oncologic risk factors (Table 2). At baseline, the extent of disease was similar in the two groups of patients: 94.4% patients treated with cetirizine and 94% not treated were at stage IV, 4.2% and 4% respectively were stage IIIC, 1.4% and 2% respectively were at stage IIIB; 19.7% and 28% respectively had CSN metastases.

**Table 2:** Univariate and multivariate analysis of oncologic risk factors in patients who had received cetirizine and in those who had not received it

Covariate	Multivariate analysis			Univariate analysis		
	HR	95% CI	p-value	HR	95% CI	p-value
*PFS*						
BRAF mut	1.635	0.9208 to 2.9015	0.0933	1.495	0.8725 to 2.5603	0.1434
CNS mtx	1.951	1.0940 to 3.4790	**0.0235**	2.159	1.3161 to 3.5423	**0.0023**
ECOG PS	4.859	2.2453 to 10.5135	**0.0001**	6.041	2.9530 to 12.3593	** < 0.0001**
Gender	1.418	0.8056 to 2.4967	0.2259	1.305	0.8221 to 2.0721	0.2587
LDH	2.058	1.2155 to 3.4841	**0.0072**	1.635	1.0384 to 2.5740	**0.0338**
M category	0.95	0.7188 to 1.2554	0.7179	0.785	0.6030 to 1.0223	0.0725
Anti-H treatment	0.595	0.3596 to 0.9853	**0.0436**	0.545	0.3467 to 0.8566	**0.0085**
*OS*						
BRAF mut	1.098	0.5510 to 2.1861	0.7913	1.066	0.5697 to 1.9959	0.8409
CNS mtx	1.647	0.8473 to 3.1998	0.1412	2.27	1.2923 to 3.9859	**0.0043**
ECOG PS	2.751	1.2144 to 6.2326	**0.0153**	4.487	2.2537 to 8.9347	** < 0.0001**
Gender	1.943	0.9641 to 3.9145	0.0632	1.426	0.8069 to 2.5196	0.2219
LDH	2.979	1.5411 to 5.7585	**0.0012**	1.851	1.0747 to 3.1873	**0.0264**
M category	0.794	0.5298 to 1.1906	0.2646	0.634	0.4236 to 0.9483	**0.0265**
Anti-H treatment	0.392	0.2092 to 0.7328	**0.0034**	0.404	0.2340 to 0.6987	**0.0012**

**Fig. 1:**
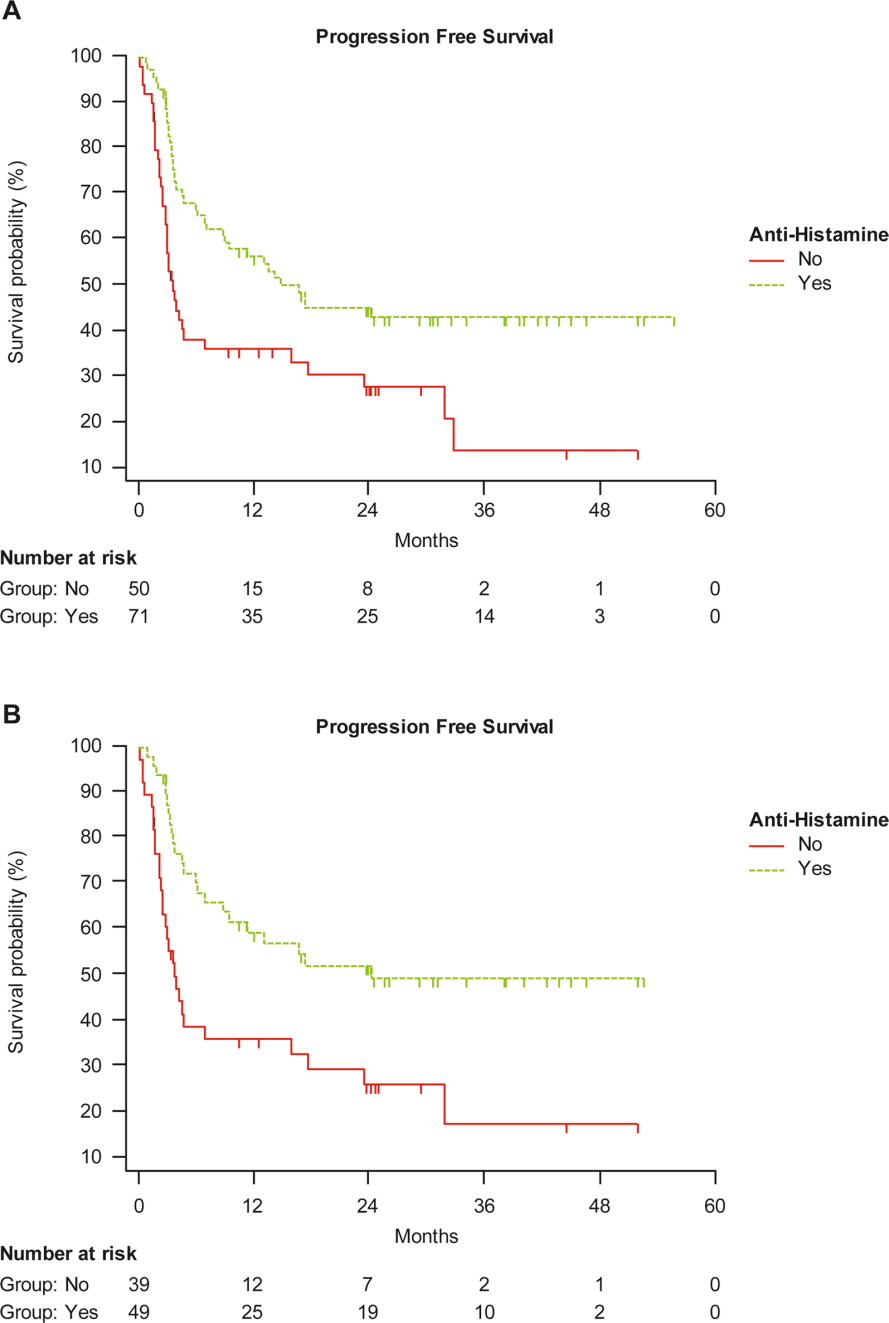
Progression-free survival in **A** the overall study population and **B** naïve patients, either receiving cetirizine or not

PFS was significantly longer in patients treated with cetirizine in comparison with those not receiving cetirizine within the total study population (mean PFS was 28 vs 15 months, HR 0.47, 95% CI: 0.29–0.76; p = 0.0023), and within naïve patients (30 vs 15 months, HR 0.40, 95% CI: 0.23–0.72; p = 0.0021) (Fig. 1A, B). No significant difference in PFS was found for pretreated patients, whether receiving cetirizine or not.

**Fig. 2:**
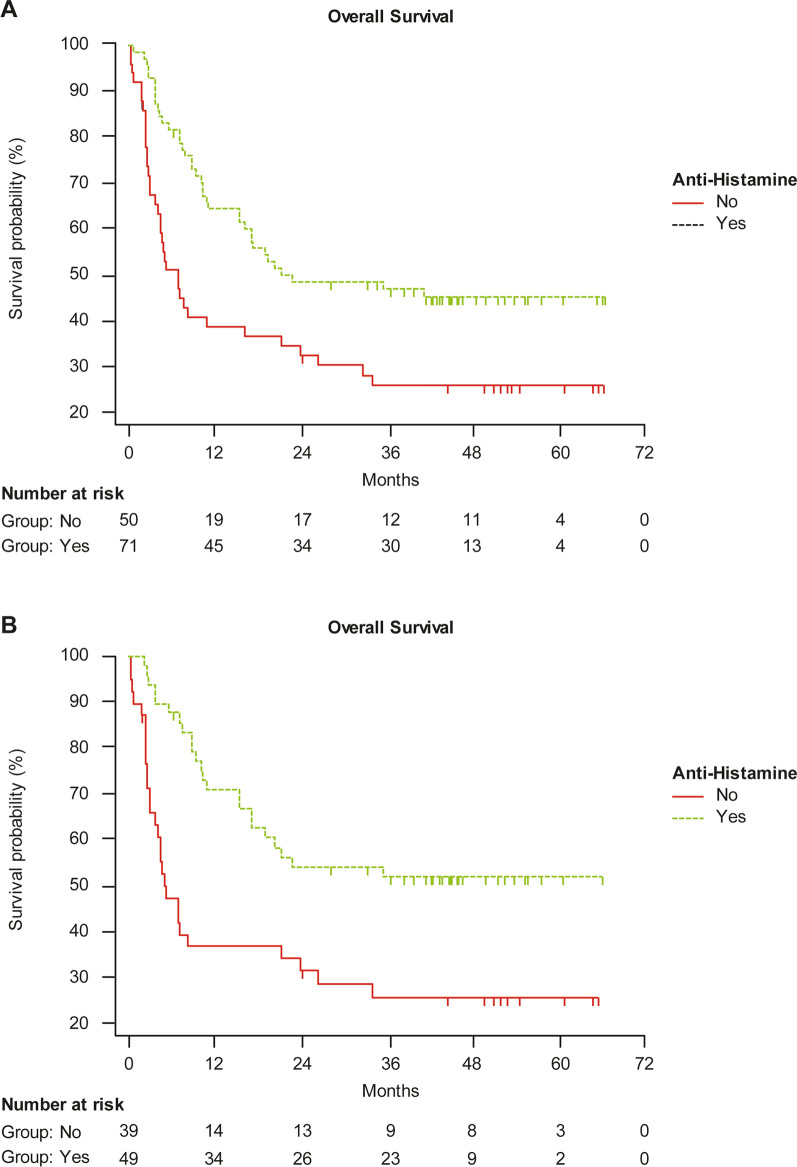
Overall survival in **A** the overall study population and **B** naïve patients, either receiving cetirizine or not

A significantly longer OS was reported for patients receiving cetirizine concomitantly with the anti-PD-1 agent in comparison with those not receiving cetirizine, within the whole study population (mean OS was 36 vs 23 months, HR 0.48, 95% CI: 0.29–0.78; p = 0.0032), and within the naïve group (mean OS was 40 vs 22 months, HR 0.37, 95% CI: 0.20–0.67; p = 0.001) (Fig. 2A, B). OS was not significantly different in pretreated patients either with or without cetirizine.

The administration of cetirizine concomitantly with the anti-PD-1 agent was significantly associated with ORR and DCR in the overall study population and the naïve population (both variables for both groups, p < 0.05), but not in the pretreated population.

### Transcriptomic analysis

Overall, blood samples were obtained from the same 120 patients before and after immunotherapy. Overall, 210 blood samples were obtained for the transcriptomic analysis. At baseline, 49 samples were gathered from patients receiving only the anti-PD-1 agent (10 pretreated and 39 naïve), and 67 from patients receiving cetirizine concomitantly with the anti-PD-1 agent (18 pretreated and 49 naïve). After 3 months of treatment with an anti-PD-1 agent or at discontinuation, 34 samples were obtained from subjects not receiving cetirizine (9 pretreated and 25 naïve) and 60 from subjects receiving cetirizine (18 pretreated and 42 naïve).

**Fig. 3:**
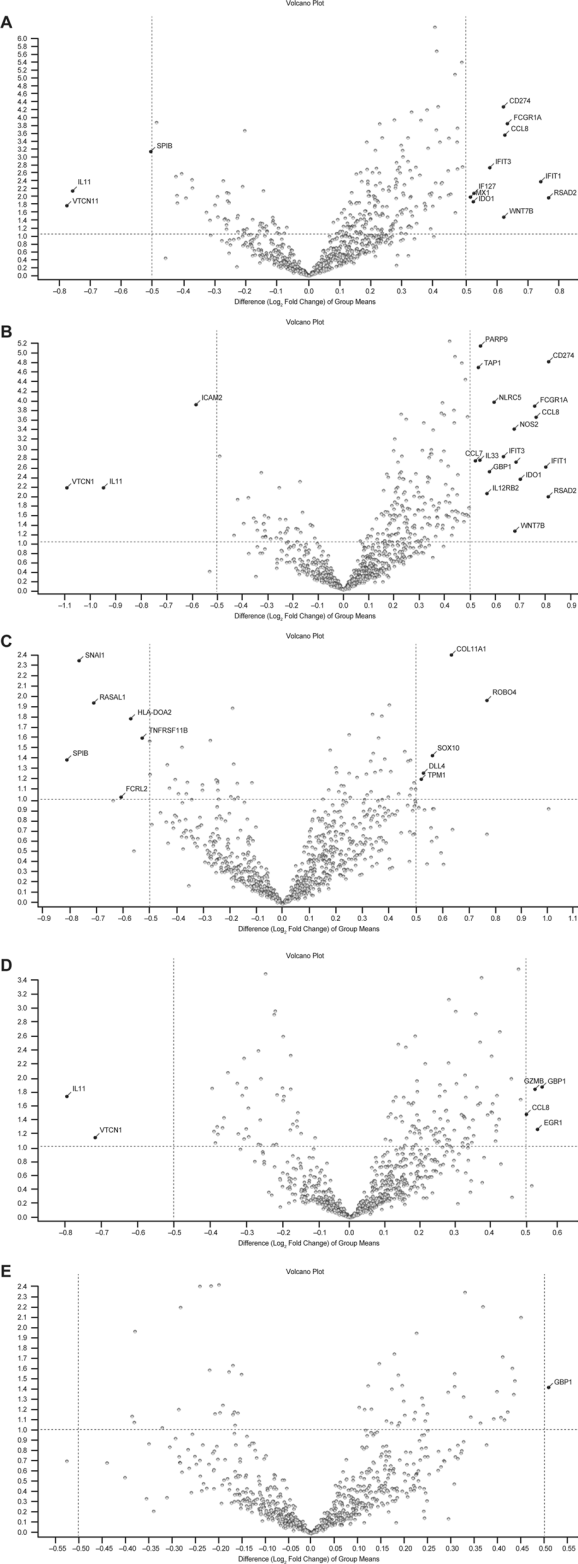
Transcritomic analysis of samples obtained at baseline and after treatment with anti-PD-1 agent. **A** all patients receiving concomitant cetirizine; **B** naïve patients receiving concomitant cetirizine; **C** patients pretreated with anti-CTLA4 agent and receiving concomitant cetirizine; **D** all patients not receiving cetirizine; **E** naïve patients not receiving cetirizine. p values are reported on the Y axis; values reported over the orizontal dotted line are significant

**Fig. 4:**
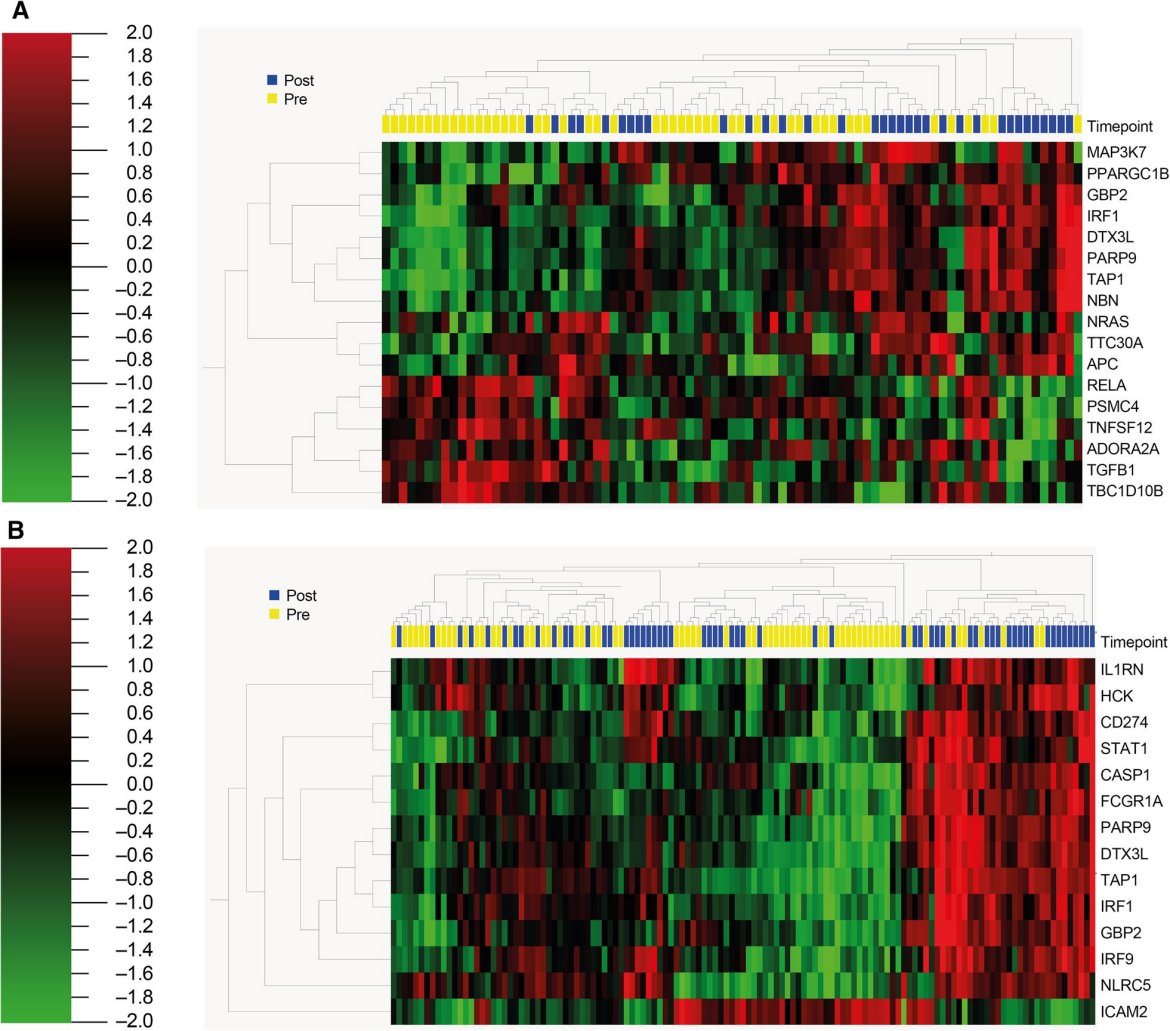
Heatmap representation of analysis on patients who had received cetirizine (**A**) and those who had not received it (**B**)

The gene expression of the high-affinity immunoglobulin gamma Fc receptor I (*FCGR1A/CD64*), C–C motif chemokine 8 (*CCL8*), interferon-induced antiviral RNA-binding protein (*IFIT1*), IFN-induced antiviral protein (*IFIT3*), and interferon-inducible antiviral protein (*RSAD2*) was increased after the treatment in comparison with baseline in the overall study population receiving cetirizine (Fig. 3A), and in naïve patients receiving cetirizine (Fig. 3B). In contrast, it was not increased in patients pretreated with ipilimumab and receiving cetirizine (Fig. 3C) and in patients not receiving cetirizine (Fig. 3D). Gene expression was unchanged after treatment also in patients who did not receive cetirizine even if naïve to checkpoint inhibitors (Fig. 3E). Genes expressed in samples from patients receiving cetirizine and those not receiving it are described in Fig. 4 and in Additional file 1: Table S1.

Finally, it was found that the expression of *FCGR1A* positively correlated with the expression of genes linked to the interferon pathway, such as *CCL8* (rho = 0.32; p = 0.0111)*, IFIT1* (rho = 0.29; p = 0.0229)*, IFIT3* (rho = 0.57; p < 0.0001)*, IFI27 (*rho = 0.42; p = 0.008), MX1 (rho = 0.26; p = 0.0383) *and RSAD2* (rho = 0.43; p = 0.0005).

## Discussion

In this retrospective study on 121 patients with histologically confirmed metastatic melanoma at stage IIIb–IV, we found that using cetirizine in concomitance with anti-PD-1 immunotherapy was associated with improved PFS, OS, ORR and DCR in subjects naïve to checkpoint inhibitors. Our results are in agreement with findings published by Li et al. (2022); we cannot compare the role of histamine levels as this data is not available, yet, and will be assessed later to further explore the subject. In addition, the transcriptomic analysis showed that patients receiving cetirizine and naïve to anti-PD-1 had a different gene expression after the immunotherapy compared to baseline. Overall, our observations suggest that concomitant cetirizine therapy may enhance the efficacy of anti-PD-1 agents, through the IFN-γ pathway promoting the M1 polarization of TAMs. We had no rationale for selecting cetirizine as a possible promoter of tumoricidal TAM polarization excepting our clinical observation of benefits in treated patients. Our results remain a provoking issue, with many open questions.

*FCGR1A*/CD64 binds the FC portion of IgG with high affinity and functions during early immune responses. It is constitutively found only on macrophages and monocytes and is a specific marker of M1 macrophages (UniProt https://www.uniprot.org/uniprot/P12314). CCL8 is a chemotactic factor that attracts monocytes, and it is involved in cellular responses to IFN-γ (UniProt https://www.uniprot.org/uniprot/P80075), as well as IFIT1, IFIT3, and RSAD2. *FCGR1A*/CD64 expression was increased after the treatment period, suggesting a polarization of TAMs to the M1 phenotype. Activation of the IFN-γ pathway was associated, as shown by the increased expression of *CCL8, IFIT1, IFIT3, RSAD2, IFI27, MX1* in our samples.

It is possible that the M1 polarization of macrophages is a mechanism responsible for the improved outcomes of immunotherapy in the subjects receiving concomitant cetirizine. Cetirizine is known to have an antihistamine activity and anti-inflammatory effects and enhance the production of IFN-gamma by peripheral blood monocytes [16]. Although this is a retrospective study, our clinical observation of a correlation between improved outcomes and concomitant cetirizine stands for a favorable drug interaction where increased macrophage killing phenotype in the tumor microenvironment has a beneficial effect potentiating the immunotherapy. Indeed, the expression of *CCL8, IFIT1, IFIT3, IFI27, MX1,* and *RSAD2* has previously been considered favorably prognostic in several types of cancer [4, 6, 8, 20].

On the contrary, we observed that the outcomes of treatment with anti-PD-1 in subjects previously treated with ipilimumab were not different in those receiving cetirizine and in those not receiving it. In pretreated patients, we could not demonstrate a change in the expression of *FCGR1A*/CD64 and genes involved in the IFN-γ pathway after the treatment period. It is possible that this result is only due to the low sample size. We can also speculate that a very aggressive tumor environment, such as in the presence of secondary resistance, may induce further mechanisms of resistance, which would block the activity of cetirizine on macrophages, along with a block of the antitumor effect of immunotherapy. Anyway, further investigation is necessary for interpretation.

In conclusion, this retrospective study suggests that M1 macrophage polarization may be induced by cetirizine and that this effect may synergize with the immunotherapy of advanced melanoma with anti-PD-1 agents.

## Supplementary Information

**Additional file 1.** **Supplementary table 1.** Description of genes expressed either with cetirizine administration or without cetirizine in the overall population.

## Data Availability

The samples were collected from July 2014 to June 2018. Data related to this article are available from the corresponding author, upon reasonable request.

## Abbreviations

*CCL8*: C–C motif chemokine 8
CR: Complete response
DCR: Disease control rate
*FCGR1A/CD64*: High-affinity immunoglobulin gamma Fc receptor I
*IFIT1*: Interferon-induced antiviral RNA-binding protein
MAF: Macrophage-activating factor
OS: Overall survival
PD: Progressive disease
PFS: Progression-free survival
PR: Partial response
*RSAD2*: Interferon-inducible antiviral protein
SD: Stable disease
TAM: Tumor-associated macrophages

## Data accessibility

Data and material have been deposited and are publicly available at 10.5281/zenodo.6340437.
