# Supplementary material for: Concomitant medication of cetirizine in advanced melanoma could enhance anti-PD-1 efficacy by promoting M1 macrophages polarization
Source: J Transl Med. 2022 Sep 30;20:436. doi: 10.1186/s12967-022-03643-w (PMC9523893; doi:10.1186/s12967-022-03643-w)
Supplement: Supplementary file 1 — Additional file 1. Supplementary table 1. Description of genes expressed either with cetirizine administration or without cetirizine in the overall population. [file 12967_2022_3643_MOESM1_ESM.docx]

**Table S1.** Description of genes expressed either with cetirizine administration or without cetirizine in the overall population

| **Gene** | **Protein full name** | **Activity** | **Link** |
| --- | --- | --- | --- |
| **With concomitant cetirizine** | | | |
| *CCL8* | C-C motif chemokine 8 | **Chemotactic factor** that attracts monocytes, lymphocytes, basophils and eosinophils. | <https://www.uniprot.org/uniprot/P80075> |
| *CD274/PDL1* | Programmed cell death 1 ligand 1 | Plays a critical role in induction and maintenance of immune tolerance to self | <https://www.uniprot.org/uniprot/Q9NZQ7> |
| *FCGR1A/CD64* | High affinity immunoglobulin gamma Fc receptor I | High affinity receptor for the Fc region of immunoglobulins gamma. **Functions in both innate and adaptive immune responses.** | <https://www.uniprot.org/uniprot/P12314> |
| *IFIT1* | Interferon-induced protein with tetratricopeptide repeats 1 | **Interferon-induced antiviral RNA-binding protein** | <https://www.uniprot.org/uniprot/P09914> |
| *IFIT3* | Interferon-induced protein with tetratricopeptide repeats 3 | **IFN-induced antiviral protein** | <https://www.uniprot.org/uniprot/O14879> |
| *RSAD2* | Radical S-adenosyl methionine domain-containing protein 2 | **Interferon-inducible antiviral protein** which plays a major role in the cell antiviral state induced by type I and type II interferon | <https://www.uniprot.org/uniprot/Q8WXG1> |
| *IDO1* | Indoleamine 2,3-dioxygenase 1 | Involved in the peripheral immune tolerance | <https://www.uniprot.org/uniprot/P14902> |
| *IL11* | Interleukin-11 | Cytokine that stimulates the proliferation of hematopoietic stem cells and megakaryocyte progenitor cells and induces megakaryocyte maturation resulting in increased platelet production. Also promotes the proliferation of hepatocytes in response to liver damage. | <https://www.uniprot.org/uniprot/P20809> |
| *SPIB* | Transcription factor Spi-B | Sequence specific transcriptional activator which binds to the PU-box, a purine-rich DNA sequence (5'-GAGGAA-3') that can act as a lymphoid-specific enhancer. | <https://www.uniprot.org/uniprot/Q01892> |
| *VTCN1* | V-set domain-containing T-cell activation inhibitor 1 | **Negatively regulates T-cell-mediated immune response by inhibiting T-cell activation,** proliferation, cytokine production and development of cytotoxicity. When expressed on the cell surface of tumor macrophages, plays an important role, together with regulatory T-cells (Treg), in the suppression of tumor-associated antigen-specific T-cell immunity. Involved in promoting epithelial cell transformation. | <https://www.uniprot.org/uniprot/Q7Z7D3> |
| *WNT7B* | Wnt-7b | Ligand for members of the frizzed family of seven transmembrane receptors that functions in the canonical Wnt/beta-catenin signaling pathway (PubMed: 30026314). | https://www.uniprot.org/uniprot/P56706 |
| *IFI27* | Interferon alpha-inducible protein 27, mitochondrial | Part of the signaling pathways that lead to apoptosis (PubMed: 18330707, PubMed: 27673746; PubMed: 24970806). **Involved in type-I interferon-induced apoptosis** characterized by a rapid and robust release of cytochrome C from the mitochondria and activation of BAX and caspases 2,3,6,8 and 9 (PubMed: 18330707, PubMed: 27673746). Also functions in TNFSF10-induced apoptosis (PubMed: 24970806). | https://www.uniprot.org/uniprot/P40305 |
| *MX1* | Interferon-induced GTP-binding protein Mx1 | Interferon-induced dynamin-like GTPase with antiviral activity against a wide range of RNA viruses and some DNA viruses. Its target viruses include negative-stranded RNA viruses and HBV through binding and inactivation of their ribonucleocapsid. Inhibits influenza. | https://www.uniprot.org/uniprot/P20591 |
| **Without concomitant cetirizin** | | | |
| *IL11* | Interleukin-11 | Cytokine that stimulates the proliferation of hematopoietic stem cells and megakaryocyte progenitor cells and induces megakaryocyte maturation resulting in increased platelet production. Also promotes the proliferation of hepatocytes in response to liver damage. | <https://www.uniprot.org/uniprot/P20809> |
| *VTCN1* | V-set domain-containing T-cell activation inhibitor 1 | Negatively regulates T-cell-mediated immune response by inhibiting T-cell activation, proliferation, cytokine production and development of cytotoxicity. When expressed on the cell surface of tumor macrophages, plays an important role, together with regulatory T-cells (Treg), in the suppression of tumor-associated antigen-specific T-cell immunity. Involved in promoting epithelial cell transformation. | <https://www.uniprot.org/uniprot/Q7Z7D3> |
| *GBP1* | Guanylate-binding protein 1 | Hydrolyzes GTP to GMP in 2 consecutive cleavage reactions | <https://www.uniprot.org/uniprot/P32455> |
| *GZMB* | Granzyme B | Abundant protease in the cytosolic granules of cytotoxic T-cells and NK-cells which activates caspase-independent pyroptosis when delivered into the target cell through the immunological synapse |  |
| *CCL8* | C-C motif chemokine 8 | Chemotactic factor that attracts monocytes, lymphocytes, basophils and eosinophils. | <https://www.uniprot.org/uniprot/P80075> |
| *EGR1* | Early growth response protein 1 | Transcriptional regulator. Regulates the transcription of numerous target genes, and thereby plays an important role in regulating the response to growth factors, DNA damage, and ischemia. | <https://www.uniprot.org/uniprot/P18146> |
